# Identifying Facilitators and Barriers to Implementation of AI-Assisted Clinical Decision Support in an Electronic Health Record System

**DOI:** 10.1007/s10916-024-02104-9

**Published:** 2024-09-18

**Authors:** Joseph Finkelstein, Aileen Gabriel, Susanna Schmer, Tuyet-Trinh Truong, Andrew Dunn

**Affiliations:** 1 Department of Biomedical Informatics, University of Utah, 421 Wakara Way, Rm. 2028, Salt Lake City, UT 84108 USA; 2 Department of Case Management, Mount Sinai Health System, New York, NY USA; 3 Division of Hospital Medicine, Icahn School of Medicine at Mount Sinai, New York, NY USA

**Keywords:** Artificial Intelligence, Clinical Decision Support, Electronic Health Record, Implementation Science, Socio-Technical Factors

## Abstract

Recent advancements in computing have led to the development of artificial intelligence (AI) enabled healthcare technologies. AI-assisted clinical decision support (CDS) integrated into electronic health records (EHR) was demonstrated to have a significant potential to improve clinical care. With the rapid proliferation of AI-assisted CDS, came the realization that a lack of careful consideration of socio-technical issues surrounding the implementation and maintenance of these tools can result in unanticipated consequences, missed opportunities, and suboptimal uptake of these potentially useful technologies. The 48-h Discharge Prediction Tool (48DPT) is a new AI-assisted EHR CDS to facilitate discharge planning. This study aimed to methodologically assess the implementation of 48DPT and identify the barriers and facilitators of adoption and maintenance using the validated implementation science frameworks. The major dimensions of RE-AIM (Reach, Effectiveness, Adoption, Implementation, Maintenance) and the constructs of the Consolidated Framework for Implementation Research (CFIR) frameworks have been used to analyze interviews of 24 key stakeholders using 48DPT. The systematic assessment of the 48DPT implementation allowed us to describe facilitators and barriers to implementation such as lack of awareness, lack of accuracy and trust, limited accessibility, and transparency. Based on our evaluation, the factors that are crucial for the successful implementation of AI-assisted EHR CDS were identified. Future implementation efforts of AI-assisted EHR CDS should engage the key clinical stakeholders in the AI tool development from the very inception of the project, support transparency and explainability of the AI models, provide ongoing education and onboarding of the clinical users, and obtain continuous input from clinical staff on the CDS performance.

## Introduction

Clinical decision support (CDS) systems are rapidly emerging as a solution to managing the abundance of medical data and delivering patient-specific recommendations to healthcare providers to assist with diagnosis and treatment [1]. CDS comprises both computerized and non-computerized tools and interventions that provide support at one or more stages of the decision-making process including alerting, interpreting, critiquing, assisting, diagnosing, and managing [2]. Studies have shown that CDS systems are effective in improving areas of healthcare delivery such as patient safety, clinical management, and cost containment [3–5]. CDS tools are often integrated with electronic health records (EHR) [6]. The widespread adoption of EHR systems has promoted the rise to prominence of CDS in healthcare delivery [7].

Recent advancements in computing have led to the development of artificial intelligence (AI) enabled healthcare technologies [8–10]. AI leverages the vast amount of data amassed from several sources, including EHR, medical imaging, laboratory data, and biosensors to forecast a clinical state, such as a diagnosis, result, or risk for CDS [11]. Healthcare providers have utilized AI -assisted CDS to guide their clinical decision-making for rare diseases, cancer, cardiovascular diseases, diabetes, sepsis, neurological diseases, and chronic obstructive pulmonary disease [12–18]. While these studies demonstrate the benefits of AI-assisted CDS on a wide range of complex medical issues, the integration of these tools brings forth a unique set of challenges including the lack of transparency in the development of the AI model or the “black box” phenomenon, workflow interference, and negative attitudes towards the use of AI [19, 20]. Understanding these barriers is imperative as the successful adoption of AI-based CDS tools may be jeopardized if they are not addressed [21–23]. The majority of previous studies focused on the accuracy of ML models, potential sources of bias, fairness, transparency, technical challenges of integrating AI-assisted CDS into commercial EHR, and impact on clinical care [24–28]. With the rapid proliferation of AI-assisted CDS, came the realization that lack of careful consideration of socio-technical issues surrounding the implementation and maintenance of these tools can result in unanticipated consequences, missed opportunities, and suboptimal uptake of these potentially useful technologies [29–32].

At the Mount Sinai Hospital System (MSHS), a number of internally developed AI-assisted CDS technologies were integrated into Epic EHR and made available to healthcare providers and administrative staff. Among these technologies is a 48-h discharge prediction tool (48DPT), which utilizes a novel machine learning algorithm to predict the discharge date for hospitalized patients using discrete clinical data from Epic EHR. An accurate prediction of patient discharge timeline allows timely engagement of clinical staff, patients, and families in the discharge planning process which is believed to be a crucial aspect of successful transitions of care [33, 34]. The hospitalized patient care plans are reviewed daily at the interdisciplinary rounds (IDR) with collaborative input from a range of hospital professionals including physicians, nurses, social workers, physical and occupational therapists, and pharmacists [35]. While the 48DPT is primarily used by the hospitalist service as part of interdisciplinary rounds, its overall adoption, acceptance, and perceived impact remain unclear.

Research on dissemination and implementation (DI) has shown that if the sociotechnical context of an intervention workflow is not taken into consideration, evidence of efficacy alone will not be sufficient to encourage acceptance and adoption [36, 37]. In DI research, conceptual frameworks are used to improve the "generalizability and interpretability of research findings" [38]. These frameworks are used to aid in understanding the success or failure of the implementation of an intervention as well as identify variables that may impact efficacy [39]. One of the most commonly used DI frameworks is RE-AIM (Reach, Effectiveness, Adoption, Implementation, Maintenance). In a variety of clinical, community, and business contexts, the RE-AIM framework has been utilized to address a broad range of populations, settings, and health challenges [40]. The RE-AIM framework was developed to address the challenges of applying the findings from scientific studies to practical applications and health policy [41]. The five dimensions of RE-AIM serve as an in-depth tool to analyze the development, adoption, and assessment of implementation strategies. RE-AIM provides actionable insights by defining the target audience whose health or behaviors will benefit (Reach), identifying the critical elements influencing the desired outcomes (Effectiveness), outlining relevant aspects of the delivery environment and staff (Adoption), evaluating adherence to established protocols in intervention delivery (Implementation), understanding the conditions that may impact long-term adoption of the intervention [42]. The Consolidated Framework for Implementation Research (CFIR) is a widely used framework that offers a comprehensive taxonomy of operationally defined constructs for understanding the barriers and facilitators that influence implementation success [38]. The CFIR framework consists of five major domains with each domain containing sub-domains and constructs: intervention characteristics, outer setting, inner setting, individuals, and process. CFIR constructs have been used to help inform the design, evaluation, and improvement of implementation strategies [38, 39].

The RE-AIM framework has been successfully used for the evaluation of EHR CDS implementation and translation of healthcare informatics interventions into routine clinical care [43, 44]. Recent studies demonstrated the high potential of the CFIR framework in supporting the implementation of new EHR functionalities while accounting for inner and outer settings [45, 46]. The combined application of the RE-AIM and CFIR frameworks for implementation assessment of clinical informatics programs has been shown to provide the most comprehensive insights on barriers and facilitators affecting the program implementation and to support effective organizational optimization [45, 47, 48]. There is a lack of systematic implementation science studies assessing the implementation of AI-assisted EHR CDS in routine clinical practice. The aim of this study was to methodologically assess the implementation of the 48-h Discharge Prediction Tool and identify the barriers and facilitators of adoption and maintenance using the RE-AIM and CFIR frameworks.

## Methods

### Study Design

We conducted semi-structured interviews with a participant sample comprised of healthcare workers who represent clinical and administrative stakeholders with a wide range of roles, responsibilities, and years of experience at the Mount Sinai Hospital System (MSHS) in New York City. A purposive sample was recruited from participating hospital units until information saturation or no new relevant information could be identified from the collected data [49]. 24 participants consented and enrolled in the study (See Table 1). The semi-structured interview questions were guided by the Reach, Efficacy, Adoption, Implementation, and Maintenance (RE-AIM) framework. Questions that pertained to unit-based leadership roles (case manager, social worker, nurse manager, unit medical director, and clinician roles (attending, hospitalist, intern/resident, nurse practitioner, physician assistant were included as part of the Reach and Adoption domains (See Appendix 1: Moderator Guide). The interviews were administered by two trained researchers using video conference software. Each interview was approximately 30 min in duration. The responses to each open-ended interview question were transcribed in Microsoft Word. The study protocol has been approved by the Program of the Protection of Human Subjects (PPHS) at the Icahn School of Medicine at Mount Sinai (STUDY-20–01955).

**Table 1:** Criteria for Valence/Strength ratings for CFIR constructs

Rating	Criteria	Valence ( ±)	Strength (0, 1, 2)
-2	The construct is a negative influence in the organization, an impeding influence in work processes, and/or an impeding influence in implementation efforts The majority of interviewees (at least two describe explicit examples of how the key or all aspects (or the absence) of a construct manifests itself in a negative way	Negative	Strong
-1	The construct is a negative influence in the organization, an impeding influence in work processes, and/or an impeding influence in implementation efforts. Respondents make general statements about the construct manifesting in a negative way but without concrete examples: 1) The construct is mentioned only in passing or at a high level without examples or evidence of actual, concrete descriptions of how that construct manifests 2) There is a mixed effect of different aspects of the construct but with a general overall negative effect 3) There is sufficient information to make an indirect inference about the generally negative influence; and/or 4) Judged as weakly negative by the absence of the construct	Negative	Weak
0	A construct has neutral influence if: 1) It appears to have neutral effect (purely descriptive) or is only mentioned generically without valence 2) There is no evidence of positive or negative influence 3) Credible or reliable interviewees contradict each other 4) There are positive and negative influences at different levels in the organization that balance each other out; and/or different aspects of the construct have positive influence while others have negative influence and overall, the effect is neutral	Neutral	Mixed
1	The construct is a positive influence in the organization, a facilitating influence in work processes, and/or a facilitating influence in implementation efforts. Interviewees make general statements about the construct manifesting in a positive way but without concrete examples: 1) The construct is mentioned only in passing or at a high level without examples or evidence of actual, concrete descriptions of how that construct manifests 2) There is a mixed effect of different aspects of the construct but with a general overall positive effect; and/or 3) There is sufficient information to make an indirect inference about the generally positive influence	Positive	Weak
2	The construct is a positive influence in the organization, a facilitating influence in work processes, and/or a facilitating influence in implementation efforts The majority of interviewees (at least two describe explicit examples of how the key or all aspects of a construct manifests itself in a positive way	Positive	Strong
M	Missing Interviewee(s) were not asked about the presence or influence of the construct: or if asked about a construct, their responses did not correspond to the intended construct and were instead coded to another construct. Interviewee(s) lack of knowledge about a construct does not necessarily indicate missing data and may instead indicate the absence of the construct	N/A	N/A

### Data Analysis

The qualitative data collected from the semi-structured interviews was analyzed using a direct content analysis approach which aims to conceptually validate or expand a theoretical framework or theory [50]. Two researchers thoroughly reviewed the transcripts of each interview and extracted all relevant textual data for input into Microsoft Excel. The raw textual data was organized and deductively coded using a priori codes guided by the five RE-AIM domains: Reach, Efficacy, Adoption, Implementation, and Maintenance. Additionally, constructs and subconstructs from select CFIR domains (*intervention characteristics, inner setting, individual, process, and outcome)* were carefully reviewed and mapped onto RE-AIM interview topics (See Appendix 2) [51]. Since the 48DPT was developed and implemented as an internal CDS, the CFIR constructs from the outer setting domain were omitted from the analysis.

Each of the textual data that was coded with a CFIR construct was assigned two ratings, a valence rating (+ , -) based on the perceived positive or negative influence on the implementation of 48DPT, and a strength rating based on the perceived weak (1) strong (2) or neutral (0) influence on the implementation of the 48DPT. Valence and strength ratings were assigned to each construct based on the ratings criteria described by the CFIR framework [52]. Constructs were rated using as having a negative influence (-) were considered as a “barrier” to implementation. Constructs rated as a positive ( +) influence on the implementation of the intervention were considered a “facilitator”. The criteria for the CFIR construct ratings are outlined in Table 1.

The valence and strength ratings across all participants for each CFIR construct were reviewed and identified as a strong facilitator, weak facilitator, strong barrier, weak barrier, and neutral (mixed) based on similar methods and criteria described by Wilson and colleagues [53]. Constructs that received mostly positive valence ratings AND at least 25% of the coded textual data was assigned a strength rating of + 2 were determined to be a “Strong Facilitator”. Constructs that were assigned mostly positive valence ratings but less than 25% of the coded textual data was rated + 2 for strength was considered to be a “Weak Facilitator”. Constructs with mostly negative valence ratings AND at least 25% of the coded textual data was assigned a strength rating of -2 were determined to be a “Strong Barrier” Constructs assigned mostly negative valence ratings but less than 25% of the coded textual data received a -2 strength rating were determined to be a “Weak Barrier”.

## Results

A total of 24 participants representing six stakeholder roles including attending, hospitalist/attending, unit medical director, case manager, nurse manager, and social worker.

Participants reported a wide range of years worked at MSHS, 33% have worked for 0 – 1 year, 33% have worked 5 – 10 years, 25% have worked 2 – 4 years, and 9% have worked for more than 10 years (See Table 2).

**Table 2:** Characteristics of participants

Stakeholder Role	*n*
Attending	9
Hospitalist/Attending	4
Unit Medical Director	4
Nurse/Case Manager	3
Social Worker	4
**Total**	24
**Length of years worked**	***n***
0—1 year	8
2—4 years	6
5—10 years	8
greater than > 10 years	2
Total	24

The results are reported according to the RE-AIM dimensions with relevant CFIR constructs provided for each RE-AIM dimension.

### Reach

The Reach domain of the RE-AIM framework examines the motivations for accepting or rejecting the use of the 48DPT. Most participants across stakeholder roles were aware of the existence of the 48DPT. When asked how often the results from the 48DPT are utilized by at least one member of the interdisciplinary team on a given weekday, responses varied by stakeholder role. Among unit-based leadership participants, social workers and case managers reported daily use of the 48DPT. Nurse managers and unit medical director participants rarely used the 48DPT results. Hospitalist/Attending and attending/unit medical director participants also reported minimal use of the 48DPT on a daily basis. The self-report measure of the daily use of the 48DPT aligns with the CFIR construct, *Innovation Recipient Impact,* which aims to assess the degree to which the innovation impacts the recipients  [51]. Since the frequency of use of the 48DPT varied by stakeholder role with social workers and case manager participants reporting daily use and limited use by nurse managers, unit medical director, hospitalists, and attendings, *Innovation Recipient Impact* was determined to be both a barrier and facilitator (Mixed) of the Reach domain (See Table 3).

**Table 3:** CFIR constructs associated with barriers and facilitators to the Reach domain

RE-AIM Domain: Reach			
CFIR Construct/ Subconstruct	Construct Definition	Barrier or Facilitator	Representative Comment
Innovation Adaptability	The degree to which the innovation can be modified, tailored, or refined to fit local context or needs	Mixed (Neutral)	*“Yes, it varies by age yeah. I think it varies. I think people are using it differently based on these parameters. For some younger patients, they don’t use it so much. For more advanced age patients with more complicated history, when it is hard to predict, they might want to use it more often.”* *[CL05, Hospitalist, 13 years]*
Innovation Recipient Impact	The degree to which the innovation impacts the recipients	Mixed (Neutral)	*“Not sure how frequently other team members, doctors and nurses, use it. I only use 24–48 h discharge on because it is more accurate. Sometimes I’ll just reach out to the team instead.” [UBL05, Social Worker, 8 months]*
Tailoring Strategies	The degree to which individuals choose and operationalize implementation strategies to address barriers, leverage facilitators, and fit context	Facilitator (Weak)	*“Yes, case managers in 5N used it in KCC, specifically Dr. X. Even though the patient is medically active, Dr. X will bring up to us that the patient is ready, and they will be discussion. Not really in nursing because we focus on the Discharge Today.”* *[UBL04, Nurse Manager, 3 years]*

When asked if the use of the 48DPT varied by patient factors (e.g., age, race, disease), most participants responded that patient factors had minimal or no influence. Age, disease, and patients with a complicated medical history were reported as factors that may affect the use of the 48DPT. The reported use of the 48DPT in consideration of patient factors aligns with the *Innovation Adaptability* CFIR construct which assesses the degree to which the intervention can be modified, tailored, or refined to fit local context or needs. Use of the 48DPT may differ based on age and disease as reported by a few participants indicating the presence of the *Innovation Adaptability* construct. However, since the patient factors did not influence the remainder of the participants, *Innovation Adaptability* was considered neither a barrier nor facilitator (Mixed) to the reach domain (See Table 3).

Unit-based leadership participants were asked if there are particular clinician groups or teams that are more or less likely to utilize the 48DPT. Responses reveal that social worker and case manager participants were more likely to use the 48DPT. Case managers often used the 48DPT as a reference point to initiate the discussion of a patient who was identified as ready for discharge during interdisciplinary rounds. For social workers, the 48DPT served as a prompt to start preparations for the discharge of a patient. Nursing was reportedly less likely to use the 48DPT and preferred to use Discharge Today, an existing discharge functionality embedded into Epic EHR. The implementation of the 48DPT differed between case managers and social workers as the information generated by them resulted in separate action steps. The varying uses of the 48DPT within different stakeholder roles indicate the presence of *the Tailoring* *Strategies CFIR construct.* This construct represents how individuals select and implement strategies to overcome barriers, utilize facilitators, and match context. Findings from the interviews indicate that the presence of the *Tailoring Strategies* constructs among the stakeholder roles was modest and only associated with case managers and social workers. The construct was absent in hospitalist, attending, unit medical directors, and nurse manager participants. Therefore, *Tailoring Strategies* was determined to be a weak facilitator to the reach of the 48DPT (See Table 3).

### Efficacy

The Efficacy domain of the RE-AIM framework focuses on understanding the overall impact of the implementation process on primary and secondary outcomes. Participants were asked to share their thoughts about the impact of using the 48DPT process on specific outcomes. Assessing the impact of the 48DPT on specific outcomes aligns with the *Reflecting & Evaluating* CFIR construct. *Reflecting & Evaluating* is described as the extent to which individuals collect and discuss quantitative and qualitative data concerning implementation progress. The *Reflecting & Evaluating* construct was expanded to be assessed across five subconstructs; *Perceived effectiveness, Personal judgement, Length of Stay, Initiation of discharge process, and Clinical/Patient outcomes.*

Regarding how effective or accurate the 48DPT is in determining patients who are medically ready for discharge, unit-based leadership and clinician participants differed in their responses (*Reflecting & Evaluating: Perceived effectiveness)*. Hospitalists responded that the 48DPT was accurate 60 – 70% of the time. Social workers stated that the 48DPT is effective but not consistently accurate and case managers reported that the 48DPT was accurate within an estimated range of 60%—80%. Additional responses included a lack of training or use of the 48DPT and a preference for the Discharge Today, the functionality that existed in Epic before 48DPT implementation.

When asked to compare the accuracy of the 48DPT to their personal/clinical judgment some participants (social workers, case managers, hospitalists) thought the 48DPT was about the same or slightly better at predicting discharge than their personal/clinical judgment (*Reflecting & Evaluating: Personal judgment)*. One participant (unit medical director) thought clinical judgment was better at predicting discharge. A few social workers mentioned that the accuracy of the 48DPT result varies especially when a patient becomes medically active and the case is referred to the medical team for review. Participants were asked if the 48DPT helped decrease the Length of Stay (LOS) (*Reflecting & Evaluating: Length of stay)*. Several participants (hospitalists, attendings, case managers, and nurse manager) responded favorably explaining that it prompts the team to initiate the discharge process earlier. Unit medical directors and social workers did not think the 48DPT helped decrease LOS as social work follow-up is needed for many patients.

Participants were asked if the 48DPT helps initiate the processes needed for discharge (e.g., scheduling appointments, and communication with outpatient providers) (*Reflecting & Evaluating:* *Initiation of discharge process)*. Several participants (attendings, unit medical directors, case managers, and social workers) responded positively and thought that the 48DPT could help start the discharge process. Additionally, they reported seeing an improvement with the use of the 48DPT when asked if the 48DPT helps improve clinical care or patient outcomes (*Reflecting & Evaluating:* *Clinical/Patient Outcomes)*. These participants attributed the improvement to the ability to discuss the discharge process and start preparation in a timely manner. In contrast, two attendings did not think the clinical care or patient outcomes would improve with the use of the 48DPT based on their experience.

The responses detailed above indicate the presence of each of the subconstructs for *Reflecting & Evaluating* as a facilitator or positive influence on the effectiveness of the 48DPT implementation process. However, since several hospitalists/attending participants reported having limited experience with the 48DPT, each of the five subconstructs was determined to be a weak facilitator of the efficacy domain (See Table 4).

**Table 4:** CFIR constructs associated with barriers and facilitators to the Efficacy/effectiveness domain

RE-AIM Domain: Efficacy/effectiveness			
CFIR Construct/ Subconstruct	Construct Definition	Barrier or Facilitator	Representative Comment
Reflecting & Evaluating: Perceived effectiveness	The degree to which individuals collect and discuss quantitative and qualitative information about the success of implementation	Facilitator (Weak)	*“When accurate, it’s a great. When not, it’s not. If a patient is stable and the prediction is 24–48 h, we need to start preparing. It’s helpful when accurate.” [UBL07, Social Worker, 7 months]*
Reflecting & Evaluating: Personal judgment		Facilitator (Weak)	*“I feel like it varies and sometimes it shows 24–48 h, but the patient becomes medically active so that skews.”* *[UBL05, Social Worker, 8 months]*
Reflecting & Evaluating: Length of stay		Facilitator (Weak)	*“Yes, it starts the process earlier. We start earlier instead of waiting till the last moment.”* *[CL06, Hospitalist, 10 years]*
Reflecting & Evaluating: Initiation of discharge process		Facilitator (Weak)	*“Yes, definitely because we notify UN coordinators, so they can start scheduling appointments. If the is yellow, we start scheduling their appointments. This sort of thing.” [UBL08, Case Manager, 7 years]*
Reflecting & Evaluating: Clinical/Patient outcomes		Facilitator (Weak)	*“I think in certain cases yes. It gets the ball rolling in some cases. As I said, when mentioned to the provider, they are so overwhelmed that it reminds them.”* *[UBL03, Case Manager, 2–4 years]*
Relative advantage: Familiarity with Discharge Today	The degree to which the innovation is better than other available innovations or current practice	Barrier (Weak)	*“They are completely different. Discharge Today is entered by the provider and there is a social aspect. 48DPT only uses medical information to predict the discharge.”* *[CL06, Hospitalist, 10 years]* *Discharge Today was already redundant to IDRs. Now, we have this 3rd on top of it* *[CL01, Hospitality/Attending, 6 years]*
Relative advantage: Perceived value compared to Discharge Today		Barrier (Weak)	*“Discharge Today—I feel that it’s useful, but I don’t fill it out by myself. I ask the residents and PH to do it. I feel that having the IDRs is enough. Filling this out is additional work. I think that there are a lot of delays when patients are leaving. They are still hanging out for some time for transportation reason or anything else”* *[CL02, Hospitalist, 4 years]*

The *Relative Advantage* construct aims to assess the performance of an intervention in comparison to current practices or alternate solutions [38]. The *relative advantage construct *was expanded into two subconstructs, *Familiarity with Discharge Today* and *Perceived Value compared to Discharge Today,* to examine the performance of the 48DPT compared to Discharge Today (DT), an embedded CDS tool available in Epic EHR.

Participants were asked about their awareness and experience with the DT *(Relative advantage: Familiarity with Discharge Today).* All participants reported being aware of the existence of the “Discharge Today” field in Epic before the study. Hospitalist, attending, unit medical director, and nurse manager participants preferred using DT in their clinical workflow while social worker and case manager participants preferred the 48DPT. These responses suggest that awareness of the DT tool is a negative influence, or barrier, to the implementation of the 48DPT. The *Relative advantage: Familiarity with Discharge Today* subconstruct was identified as a weak barrier because familiarity with the DT tool did not deter the adoption of the 48DPT by social workers and case managers.

When participants were asked which of the two tools was more beneficial, if the tools were redundant, or whether they each provided unique information, responses across the stakeholder roles were mixed *(Relative advantage: Perceived value compared to Discharge Today)*. Attending, unit medical director, case manager, and nurse manager participants thought that information from the 48DPT differed from the DT tool. In contrast, Hospitalist and Social worker participants stated that information from 48DPT overlaps with information from DT and interdisciplinary rounds (IDR). Two hospitalists responded that interdisciplinary rounds (IDRs) are sufficient in determining which patients are medically ready for discharge and that both the DT tool and the 48DPT are redundant. These findings indicate several participants found the information generated by the 48DPT to be redundant with the information from the DT and IDRs which can negatively influence implementation. However, this did not deter participants from adopting the 48DPT. Therefore, the *Relative advantage: Perceived value compared to the Discharge Today* subconstruct was rated as a weak barrier to the implementation of the 48DPT (See Table 4).

### Adoption

The Adoption domain examines 48DPT usage patterns at the individual and unit levels, as well as the acceptance of participants across medical specialties and levels of clinical experience. Understanding the intended use of an intervention aligns with the *Adapting* construct which measures the degree to which individuals modify the innovation and/or the Inner Setting for optimal fit and integration into work processes [38]. The *Adapting* construct was expanded into two subconstructs to assess the *Individual intended use* and the *Unit-based intended use* of the 48DPT. When asked about the use of the 48DPT, most participants responded that the 48DPT is used as originally intended *(Adapting: Individual Intended Use).* A few social worker participants were unsure if the 48DPT was being used as intended. We asked hospitalists if the use of 48DPT varied by unit and one stated that the use of the 48DPT differed across hospital units depending on the role and integration with IDRs *(Adapting: Unit-based intended use)*.

The findings discussed above indicate the presence of both *Adapting* subconstructs as a facilitator or positive influence on the implementation and adoption of the 48DPT. The *Adapting: Individual Intended Use* and *Adapting: Unit-based intended use* subconstructs were identified as weak facilitators of implementation because several hospitalists had awareness of the 48DPT but did not have experience using the tool (See Table 5).

**Table 5:** CFIR constructs associated with barriers and facilitators to the Adoption domain

RE-AIM Domain: Adoption			
CFIR Construct/ Subconstruct	Construct Definition	Barrier or Facilitator	Representative Comment
Adapting: Individual intended Use	The degree to which individuals modify the innovation and/or the Inner Setting for optimal fit and integration into work processes	Facilitator (Weak)	*“Not sure. I don’t rely on it so much. Not sure if the medical team relies on it."* *[UBL05, Social Worker, 8 months]*
Adapting: Unit-based intended use		Facilitator (Weak)	*“Yes, like 5N where it was rolled out initially, it’s more robust. Not sure about other people. It used to be a Case Manager but now we want it to be more integrated in IDRs.”* *[CL06, Hospitalist, 10 years]*
Knowledge & Beliefs: Clinician response to 48DPT	Individuals’ attitudes toward and value placed on the innovation, as well as familiarity with facts, truths, and principles related to the innovation	Facilitator (Weak)	*“More times than not they will agree that the patients will be discharged in 48 h. Sometimes it will be “no, the machine is wrong” but most times clinicians agree with it.”* *[UBL03, Case Manager, 2–4 years]*
Knowledge & Beliefs: Response to 48DPT by clinician role		Facilitator (Weak)	*“Absolutely. The algorithm strictly takes in account the medical information. For clinician it might be different but for us as social workers it is useful, but again I am not a doctor.”* *[UBL06, Social Worker, 1 year]*
Knowledge & Beliefs: Response to 48DPT by specialty or roles		Facilitator (Weak)	*“Providers have all different roles and the 48DPT might help with each role. I would say yes. It does vary. For ADS, it might be a primary, because only 1 provider is involved. When it comes to Family Med, there are different providers—residents and others. It can be a barrier. Different people can come forward and ask different questions.”* *[UBL01, Social Worker, 11 months]*
Knowledge & Beliefs: Response to 48DPT by clinician's time in practice		Barrier (Weak)	*“More experienced attendings, not every single one of them, are like “Ok, I know how to do my job”. The newer ones came at time when AI is consistently used are more used to it.”* *[UBL07, Social Worker, 7 months]*

The *Knowledge & Beliefs* CFIR construct aims to seek to examine the views and the value placed on the intervention including the familiarity with facts, truths, and principles [38]. The *Knowledge & Beliefs* were adapted to be assessed across four subconstructs; *Clinician response to 48DPT*, *Response to 48DPT by clinician role, Response to 48DPT by specialty or roles,* and *Response to 48DPT by clinician's time in practice.*

Participants in unit-based leadership roles (social workers, case managers, and unit medical directors) were asked to discuss how clinicians might react or respond if a team member announces that a discharge is predicted within the next 48 h *(Knowledge & Beliefs: Clinician response to 48DPT)*. Most unit-based leadership participants reported that team members would be accepting and welcoming to the information. They mentioned that clinician reactions to a 48DPT result may vary depending on the clinician's role *(Knowledge & Beliefs: Response to 48DPT by clinician role)* and the medical specialty *(Knowledge & Beliefs: Response to 48DPT by specialty or roles).*

These findings suggest the presence of the *Knowledge & Beliefs: Clinician response to 48DPT, Knowledge & Beliefs: Response to 48DPT by clinician role, and Knowledge & Beliefs: Response to 48DPT by specialty or role* subconstructs as a facilitator or positive influence on the implementation and adoption of the 48DPT. These subconstructs were recognized as weak facilitators of implementation since some hospitalists and attendings did not use the 48DPT regularly despite having knowledge about the existence of the tool (See Table 5).

When asked if the response to the 48DPT varied according to clinician’s time in practice, two unit-based leadership participants mentioned that newer clinicians are more likely to not be aware of the 48DPT compared to more experienced clinicians (*Knowledge & Beliefs: Response to 48DPT by clinician's time in practice).* The lack of awareness about the 48DPT by clinicians with limited time in practice was determined to be a barrier to implementation and adoption. Since the limited knowledge did not deter the adoption of the 48DPT by less experienced clinicians, the *Knowledge & Beliefs: Response to 48DPT by clinician's time in practice* subconstruct was rated as a weak barrier (See Table 5).

### Implementation

The Implementation domain of the RE-AIM framework aims to understand the enabling and predisposing factors that support the implementation of the 48DPT. Assessing the enabling and predisposing factors aligns with the *Engaging* CFIR construct. This construct refers to the degree to which individuals attract and promote involvement in the implementation process and/or the intervention [38]. The *Engaging* construct was expanded to two subconstructs to examine its presence or absence in *Individuals* and *Interdisciplinary Team Members*. Participants were asked if they were involved in developing the process for the use of the 48DPT *(Engaging: Individuals)*. Most participants responded that they were not involved in the development process. These responses reveal that the *Engaging: Individuals* subconstruct was largely absent. However, the lack of participation in the development did not hinder the adoption of the 48DPT by several participants. Therefore, the *Engaging: Individuals* subconstruct was rated as a weak barrier to the Implementation domain (See Table 6).

**Table 6:** CFIR constructs associated with barriers and facilitators to the Implementation domain

RE-AIM Domain: Implementation			
CFIR Construct/ Subconstruct	Construct Definition	Barrier or Facilitator	Representative Comment
Access to Knowledge & Information: Training and Support	The degree to which guidance and/or training is accessible to implement and deliver the innovation	Facilitator (Weak)	*“Aside from educating the front lines, that’s the key. I don’t think a lot of people are aware of it. I’d say educate the residents and the attendings.”* *[UBL02, Unit Medical Director, 10 years]*
Access to Knowledge & Information: Computer Skills		Facilitator (Strong)	*“I don’t think its super challenging. We use a lot of programs in the field. Just a brief explanation what it is like.” [UBL07, Social Worker, 7 months]*
Compatibility	The degree to which the innovation fits with workflows, systems, and processes	Mixed (Neutral)	*“Somewhat. There are more opportunities to be integrated. Now it’s more case managers. It’s very passive and it needs to be more active.”* *[CL06, Attending/UMD, 5–10 years]*
Engaging: Individuals	The degree to which individuals attract and encourage participation in implementation and/or the innovation	Barrier (Weak)	*“No, it was already introduced to us by management. It was already introduced and implemented, and I don’t know about its development.”* *[UBL08, Case Manager, 7 years]*
Engaging: Interdisciplinary Team Members		Barrier (Strong)	*“I don’t know. I don’t think anyone on my team was involved.”* *[CL04, Unit Medical Director, 7 years]*

Participants were asked about the involvement of interdisciplinary team members (e.g., case managers, nurse managers, and clinicians) in the development process of the 48DPT *(Engaging: Interdisciplinary Team Members)*. The majority of participants reported having no knowledge of the 48DPT development process or if other team members were involved. A few participants did not think that the interdisciplinary team was adequately involved. Case manager and nurse manager participants responded that the involvement of interdisciplinary team members in the development of the 48DPT was satisfactory. These findings suggest that stakeholder engagement was limited and the lack of involvement of interdisciplinary team members is a negative influence on adoption. Participants were either unaware of the development phase and the involvement of other team members or did not think the efforts to involve team members were sufficient. Therefore, the *Engaging: Interdisciplinary Team Members* subconstruct was determined to be a strong barrier to implementation (See Table 6).

The *Compatibility* CFIR construct looks at how effectively the intervention fits with current processes, systems, and practices [38]. When asked if the 48DPT was integrated well into their workflow, the responses across roles were mixed. Several participants (unit medical director, and nurse manager) responded that the 48DPT was not adopted into their workflow. These participants suggested providing training and reminders about the availability of the tool to improve integration. Some hospitalists and attendings thought that the 48DPT was somewhat integrated into their workflow due to a lack of follow-up education and efforts to increase awareness of the 48DPT after the initial rollout. A few case managers and social workers reported that the 48DPT was well-integrated. These varied responses indicate that the *Compatibility* construct did not have a positive or negative influence on the implementation domain and was determined to be neither a barrier nor a facilitator (Mixed) (See Table 6).

Participants were asked what support services and technical skills are needed by users of the 48DPT. This question corresponds with the *Access to Knowledge & Information* CFIR construct which seeks to assess the degree to which guidance and/or training are available for intervention implementation. The *Access to Knowledge & Information* construct was expanded into two subconstructs to evaluate the *Training and Support* and *Computer skills* required for the use of the 48DPT. In regard to support services, increasing awareness about the availability of the 48DPT was highlighted by several participants. Additionally, participants suggested providing training and demonstration for each member of the interdisciplinary team and improving the accuracy of the 48DPT. A few participants thought the 48DPT was intuitive to use and did not require additional training or support. These responses indicate the presence of the *Access to Knowledge & Information: Training and Support* subconstruct as a weak facilitator of the implementation domain (See Table 6).

When asked what computer skills were necessary to successfully use the 48DPT, most participants stated that basic computer skills are needed. Participants were also familiar with the general operation of the 48DPT because of their prior experience using similar programs and in their clinical workflow. These findings reveal the presence of *Access to Knowledge & Information: Computer Skills* subconstruct as a facilitator or positive influence on the implementation of the 48DPT. Because the majority of participants reported possessing the requisite technical abilities for the successful deployment of the 48DPT, this subconstruct was evaluated as a strong facilitator (See Table 6).

### Maintenance

The Maintenance domain of the RE-AIM framework investigates the degree to which the 48DPT is assimilated into organizational culture and routine practice. Examining the factors related to the success of the implementation of an intervention aligns with the *Assessing Context* CFIR construct. The *Assessing Context* construct aims to assess the extent to which individuals gather information to recognize and evaluate obstacles and enablers for implementing and delivering the innovation [38]. This construct was broadened to be examined across four subconstructs; *Perceived burden*, *Comparison of perceived benefits to burden*, *Perceived barriers,* and *Use of 48DPT over time*.

Participants were asked how much of a burden using the 48DPT*.* Eight participants responded that the use of the 48DPT did not add a burden. When asked to compare the burden of using the 48DPT to the benefits, the majority of participants responded that the benefits outweigh any burden *(Assessing Context: Comparison of perceived benefits to burden).* Findings from these responses indicate the presence of the *Assessing Context: Perceived burden* and *Assessing Context: Comparison of perceived benefits to burden* subconstructs as facilitators or positive influences on the implementation and maintenance of the 48DPT. Overall, participants did not find the 48DPT to be burdensome and agreed that the benefits of using the tool overshadowed any difficulties. Therefore, the *Assessing Context: Perceived burden* and *Assessing Context: Comparison of perceived benefits to burden* subconstructs were rated as strong facilitators of 48DPT implementation (See Table 7).

**Table 7:** CFIR constructs associated with barriers and facilitators to the Maintenance domain

RE-AIM Domain: Maintenance			
CFIR Construct/ Subconstruct	Construct Definition	Barrier or Facilitator	Representative Comment
Assessing Context: Perceived burden	The degree to which the individual(s) collect information to identify and appraise barriers and facilitators to implementation and delivery of the innovation	Facilitator (Strong)	*“I don’t think it’s a burden. It’s additional support for the providers. Sometimes it won’t agree with the providers, but you can’t take away that it’s helpful. The providers will say they will check again.” [UBL04, Nurse Manager, 3 years]*
Assessing Context: Comparison of perceived benefits to burden		Facilitator (Strong)	*“Yes, yes if it’s really accurate in predicting discharge, the benefits will outweigh the barriers. [UBL02, UMD, 10 years]*
Assessing Context: Perceived barriers		Barrier (Strong)	*“There are no barriers except if there is a way to get an answer after lunch. It is just before lunch. Sometimes when they come in the afternoon, the shift is already over and they (the patients) go yellow, you’re not able to do anything about it.”* *[UBL08, Case Manager, 7 years]* * “Because of the high turnover of IDR members and providers, they are not aware of it.”* *[CL06, Hospitalist, 10 years]*
Assessing Context: Use of 48DPT over time		Barrier (Weak)	*“I think it’s already embedded in the process for the provider. I’m not sure but that’s my observation. I think it’s been used in the same way otherwise I wouldn’t hear Dr. X bringing it up.”* *[UBL04, Nurse Manager, 3 years]*
Assessing Needs: Innovation Recipients	The degree to which the individual(s) collect information about the priorities, preferences, and needs of recipients to guide implementation and delivery of the innovation	Facilitator (Weak)	*“By educating about it, for example educating 10C, at least the team leader what it is and how to use it and how to use it during IDRs or how to use it with my own patients.” [CL04, Hospitalist, 7 years]* * “Understanding it more. Adding the psychosocial aspect. Even with disposition, it will be helpful to know where the patient comes from a facility and that they need to go back there.” [UBL07, Social Worker, 7 months]*
Available Resources	The degree to which resources are available to implement and deliver the innovation	Barrier (Weak)	*“The unit-base metrics. We do talk about it. We talk about it on daily bases.”* *[UBL06, Social Worker, 1 year]*
Goals & Feedback	The degree to which goals are clearly communicated, acted upon, and fed back to staff, and alignment of that feedback with goals	Barrier (Strong)	*“Not that I know of. I think the only time was I spoke with Dr. Y, maybe with Dr. X, a few months ago. Only then the leaders reached out to see what works or doesn’t about it.”* *[UBL03, Case Manager, 2–4 years]*

When asked about the barriers to using the 48DPT, participants most frequently mentioned the lack of awareness of the tool *(Assessing Context: Perceived barriers)*. Additional reported barriers were the lack of educational training, limited integration into IDRs, and difficulty communicating with providers. These results reveal the prevalence of the *Assessing Context: Perceived barriers* subconstruct and barriers such as lack of awareness about 48DPT pose a negative influence on adoption. Therefore, *Assessing Context: Perceived barriers* were identified as a strong barrier to the implementation of the 48DPT (See Table 7).

Participants were asked if the use of the 48DPT changed over time. Most participants responded that the use of the 48DPT has not changed since their initial use. This question was omitted for participants who reported having limited familiarity with the use of the 48DPT. These responses indicate the absence of the *Assessing Context: Use of 48DPT over time* subconstruct which is deemed as a barrier. Since a number of participants had minimal experience with the 48DPT, the *Assessing Context: Use of 48DPT over time* subconstruct was identified as a weak barrier to the implementation and maintenance of the 48DPT (See Table 7).

The *Goals & Feedback* CFIR construct refers to the degree to which objectives are explicitly stated, acted upon, and given back to team members, as well as the alignment of that feedback with goals [38]. When asked if any efforts were made to obtain feedback about the 48DPT in order to make changes, a few participants reported attempts from leaders. Most participants were not aware of any opportunities to share their feedback about the 48DPT with leaders. The findings from these responses reveal that the presence of the *Goals and feedback* construct was minimal. The limited efforts to gather feedback and address concerns about the use of 48DPT were determined to be a barrier or negative influence on the implementation process of the 48DPT. Therefore, the Goals & Feedback construct was recognized as a strong barrier (See Table 7).

The *Available Resources* CFIR construct examines the availability of resources to implement and deliver the innovation. We asked participants if any tools helped improve their use of the 48DPT (e.g., training sessions, individual feedback, unit-based metrics). The unit-based metrics and feedback and discussions with the clinical team about discharge were found to be helpful for some participants. Most participants did not report any tools that were beneficial or did not have extensive experience with the 48DPT. These results highlight the absence of the *Available Resources* construct which is deemed as a negative influence or barrier to implementation. However, the lack of accessibility to support the use of the 48DPT did not hinder the implementation and adoption. Therefore, the *Available Resources* construct was recognized as a weak barrier (See Table 7).

Participants were asked how the use of the 48DPT could be improved. This question corresponds with the *Assessing Needs: Innovation Recipients* construct which aims to examine the extent to which the individual(s) gather information about the priorities, preferences, and needs of recipients to guide the implementation and delivery of the intervention. Increasing awareness and education of the 48DPT was a frequent suggestion, especially among hospitalists, nurse managers, and social workers. Participants also suggested including consideration of social factors as part of the algorithm and improving the accuracy of the 48DPT. This question was omitted for participants who reported having minimal use of the 48DPT. Findings from these responses suggest the presence of the *Assessing Needs: Innovation Recipients* construct as a facilitator or positive influence on the implementation process. A few participants had minimal experience with the 48DPT, therefore the *Assessing Needs: Innovation Recipients* construct was rated as a weak facilitator to the implementation and maintenance.

## Discussion

The current study sought to methodologically evaluate the implementation of a AI-assisted CDS known as the 48-h discharge prediction (48DPT) which utilizes available patient EHR data to predict readiness for discharge. The RE-AIM (Reach, Effectiveness, Adoption, Implementation, and Maintenance) framework was applied in combination with the Consolidated Framework for Implementation Research (CFIR) framework to examine the factors that impact the implementation of the 48DPT. Semi-structured qualitative interviews based on the RE-AIM framework were conducted with 24 healthcare professionals consisting of clinical and administrative stakeholders with a range of roles, duties, and years employed. Guidelines from the CFIR framework informed the analysis of the qualitative interview data using a content analysis methodology. The findings identified several facilitators and barriers which were summarized and reported using the RE-AIM dimensions.

The Reach domain explores the factors related to accepting or rejecting the usage of the 48DPT at an individual level. The variation in the adoption of the 48DPT across stakeholder roles demonstrated differing perspectives, which corresponded with the CFIR construct, Innovation Recipient Impact, and was determined to be neither a facilitator nor barrier factor to implementation. While Hospitalists/Attendings described seldom use of the tool, indicating an inherent challenge, Social Workers and Case Managers reported regular use of the 48DPT, indicating a positive influence. The Capability construct emerged as a strong barrier to implementation due to the reported lack of awareness about the 48DPT. The Innovation Adaptability construct was determined as neither a barrier nor a facilitator in the study, indicating that patient variables had a minimal impact on the use of the 48DPT. The Tailoring Strategies construct was observed among Case Managers and Social workers but not in other stakeholder roles. This absence implies a weak facilitator to adjust the use of the 48DPT to meet the contextual needs of stakeholders.

The overall impact of the implementation of the 48DPT on key outcomes was examined as part of the Efficacy domain. The Reflecting & Evaluating construct was examined across five subconstructs; Perceived effectiveness, Personal judgement, Length of Stay, Initiation of discharge process, and Clinical/Patient outcomes. Each subconstruct was recognized to be a weak facilitator due to the varying perceptions of the effectiveness of the 48DPT across stakeholders. The Relative Advantage construct which compared the performance of the 48DPT with an existing (Discharge Today) was expanded into two subconstructs; Familiarity with Discharge Today and Perceived value compared to Discharge Today). Participants across stakeholder roles were aware of the Discharge Today. Participants in the Hospitalist/Attending, Attendings/Unit Medical Director, Unit Medical Director, and Nurse Manager groups favored employing Discharge Today in their clinical workflow, but participants in the Social Worker and Case Manager groups preferred using the 48DPT. Therefore, the Familiarity with Discharge Today subconstruct was identified as a weak barrier. The Perceived value compared to the Discharge Today subconstruct was determined to be a weak barrier because several participants viewed the information provided by the 48DPT to be redundant with information from the Discharge Today and Interdisciplinary Rounds, which can have a detrimental impact on implementation. These findings highlight the need for clear communication on the unique benefits of the 48DPT.

The Adoption domain investigated the usage patterns and acceptance across medical specialties at the individual and unit levels. The Adapting construct was identified as a weak facilitator at both individual and unit levels, suggesting that stakeholders generally used the 48DPT as intended. The Knowledge & Beliefs construct was examined across four subconstructs. The Clinician response to 48DPT, Response to 48DPT by clinician role, and Response to 48DPT by specialty or roles subconstructs were identified as weak facilitators to implementation revealing positive perceptions and general acceptance of the information provided by the 48DPT. Newer clinicians were reported less likely to.be aware about the 48DPT compared to clinicians with more experience, therefore the Response to 48DPT by clinician's time in practice was recognized Overall, the findings from the Adoption domain emphasize the importance of stakeholder education and awareness for successful adoption.

The Implementation domain focuses on enabling and predisposing factors related to implementation. The Engaging: Individuals and Engaging: Interdisciplinary Team Members subconstructs examined the involvement of stakeholders in the development and implementation process of the 48DPT and were identified as strong barriers. Lack of stakeholder involvement can significantly hinder the implementation and adoption. The Compatibility construct was neither a barrier nor a facilitator, which indicates the clinical workflow integration of the 48DPT varied among participants. The Access to Knowledge & Information construct, which assesses the guidance/training resources necessary for the use of 48DPT, was expanded into two subconstructs. The Training and Support subconstruct was identified as a weak facilitator due to the prevalence of the construct and range of responses including suggestions to increase awareness of 48DPT, improve the accuracy of the 48DPT, provide comprehensive training for each team member, and additional training was not necessary. Participants reported basic computer skills are needed for the use of the 48DPT which was recognized as a strong facilitator to implementation.

Lastly, the Maintenance domain investigates the factors influencing the assimilation of the 48DPT into routine practice. The Assessing Context construct which examines the extent to which feedback is gathered to understand the implementation process to improve delivery and adoption was expanded into four subconstructs. The Perceived burden and Comparison of perceived benefits to burden subconstructs demonstrated positive perceptions of the 48DPT’s benefits outweighing any perceived burden, acting as strong facilitators to implementation. The Perceived barriers subconstruct was identified as a strong barrier due to the reported lack of awareness, lack of educational training, and limited integration. The absence of the Use of 48DPT over time subconstruct was recognized as a weak barrier. The Available Resources construct was identified as a weak barrier, suggesting that despite the absence of accessible s, participants still successfully adopted the 48DPT. The Assessing Needs: Innovation Recipients construct acted as a weak facilitator, indicating the potential for improvement through increased awareness and education. The Goals & Feedback construct revealed the lack of a feedback mechanism and limited efforts to address needs and concerns about the use of 48DPT which was considered to be a strong barrier to implementation.

The combination of the RE-AIM and CFIR frameworks has been applied to assess the implementation planning process. For example, Breathewell, an internally developed intervention for asthma care, has been evaluated in an integrated healthcare organization setting [45]. Breathewell is a technology-based asthma intervention that was planned and implemented by a multi-disciplinary team of researchers and healthcare professionals at Kaiser Permanente Colorado [45]. Researchers selected the RE-AIM framework to investigate the “who, what, where, how, and when” of the implementation outcomes for the Breathewell intervention. Specific domains and constructs were chosen from the CFIR framework to understand “why” the implementation of the Breathewell intervention was successful or unsuccessful [42, 54]. Findings from the assessment highlighted the determinants that supported the diffusion or “pull” factors and the dissemination or “push” factors associated with the implementation of the intervention [42]. Push factors refer to systematic initiatives aimed at eliciting engagement from potential adopters. Pull factors refer to the preexisting perceptions, requirements, and capacities of potential adopters that motivate change and indicate successful diffusion [54].

Applying the RE-AIM and CFIR frameworks in tandem to assess the implementation of the 48DPT allowed for a comprehensive evaluation that integrated both outcome measurement and the identification of factors influencing those outcomes. The RE-AIM framework allowed for the systematic measurement of critical dimensions such as reach, effectiveness, adoption, implementation, and maintenance. These dimensions are essential for understanding the broader public health impact of an intervention. However, while RE-AIM is robust in addressing these aspects, it does not inherently explain the factors influencing these outcomes. The CFIR framework complemented RE-AIM by offering a structured way to identify and analyze the contextual and organizational factors that could act as barriers or facilitators to successful implementation. By integrating CFIR constructs, we investigated the reasons behind the success or failure of certain RE-AIM outcomes. This allowed us to gather insights into the particular context of the 48DPT implementation, including organizational culture, leadership involvement, and the fit of the 48DPT within existing workflows. The use of a dual-framework approach enabled a deeper understanding of both the effectiveness and the underlying mechanisms that influenced the implementation of the 48DPT. This allows for the development of new strategies to improve the adoption and maintenance of an AI-assisted EHR CDS tool.

The barriers and facilitators identified by the RE-AIM and CFIR frameworks can help improve the development and implementation strategies of an AI-assisted EHR CDS tool. Our findings highlighted a significant need for early engagement of stakeholders. The involvement of a diverse group of clinicians during the initial development phase ensures that the tool fits the needs of varying clinical workflows. Feedback from the end users or the clinicians for whom the tool is intended should guide the design of the CDS interface. A user-focused design approach ensures that the tool is intuitive, integrates seamlessly into existing workflows, and addresses the specific needs and preferences of the end users. Continuous training and education are essential to the successful integration of the tool. Educating clinicians on the CDS tool's overall usage, important AI principles like biases and limitations, and how these technologies can improve clinical decision-making can help empower them to use it more effectively. Establishing a feedback mechanism that continuously gathers input and suggestions from clinicians ensures that the CDS tool remains beneficial and effective. By consistently seeking and integrating user feedback, the tool can be improved over time, becoming more adaptable to the evolving needs of end users and maintaining its overall utility. Highlighting and disseminating successful examples of an AI-assisted CDS in action can greatly encourage its broader adoption. Sharing these use cases via media, presentations, and institutional rounds can help illustrate the practical benefits of the AI-assisted EHR CDS tool. This can help bolster confidence among potential users and promote a culture of innovation. Due to the 'black box' nature of AI applications, a high degree of explainability and transparency is necessary to develop trust in AI-assisted tools. Improving the tool's accuracy and providing clear explanations and visualizations of how the AI makes decisions fosters confidence in the tool. End users are more inclined to trust and integrate the tool into their clinical workflow when they can see how the AI operates internally and comprehend the reasoning behind its recommendations. Maintaining and respecting clinician autonomy in their decision-making is crucial. It is imperative to clearly describe that the AI-assisted CDS is a supplementary tool intended to complement healthcare providers' judgment, not to take its place. Long-term maintenance of the AI-assisted CDS tool requires strong and consistent support from institutional leadership. In addition to providing the infrastructure and resources necessary for adoption, leadership endorsement informs the organization as a whole about the importance of the tool. The successful implementation of an AI-assisted tool requires cultivating a culture that values innovation and continuous learning. An organization is more receptive to change when a philosophy of experimenting, learning from mistakes, and adapting to new technologies is encouraged.

**Fig. 1:**
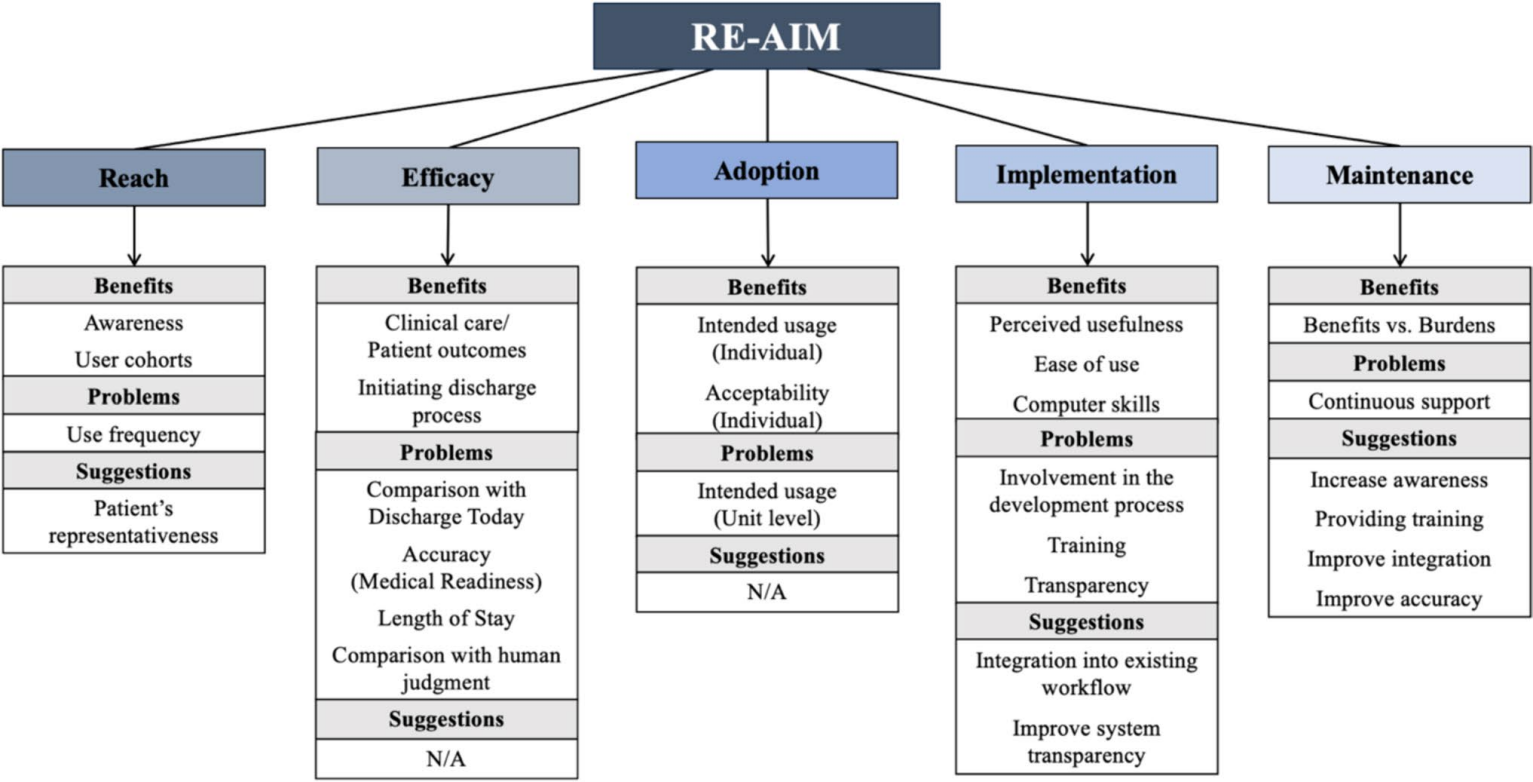
Concept map of RE-AIM dimensions and themes associated with benefits, problems, and suggestions

**Fig. 2:**
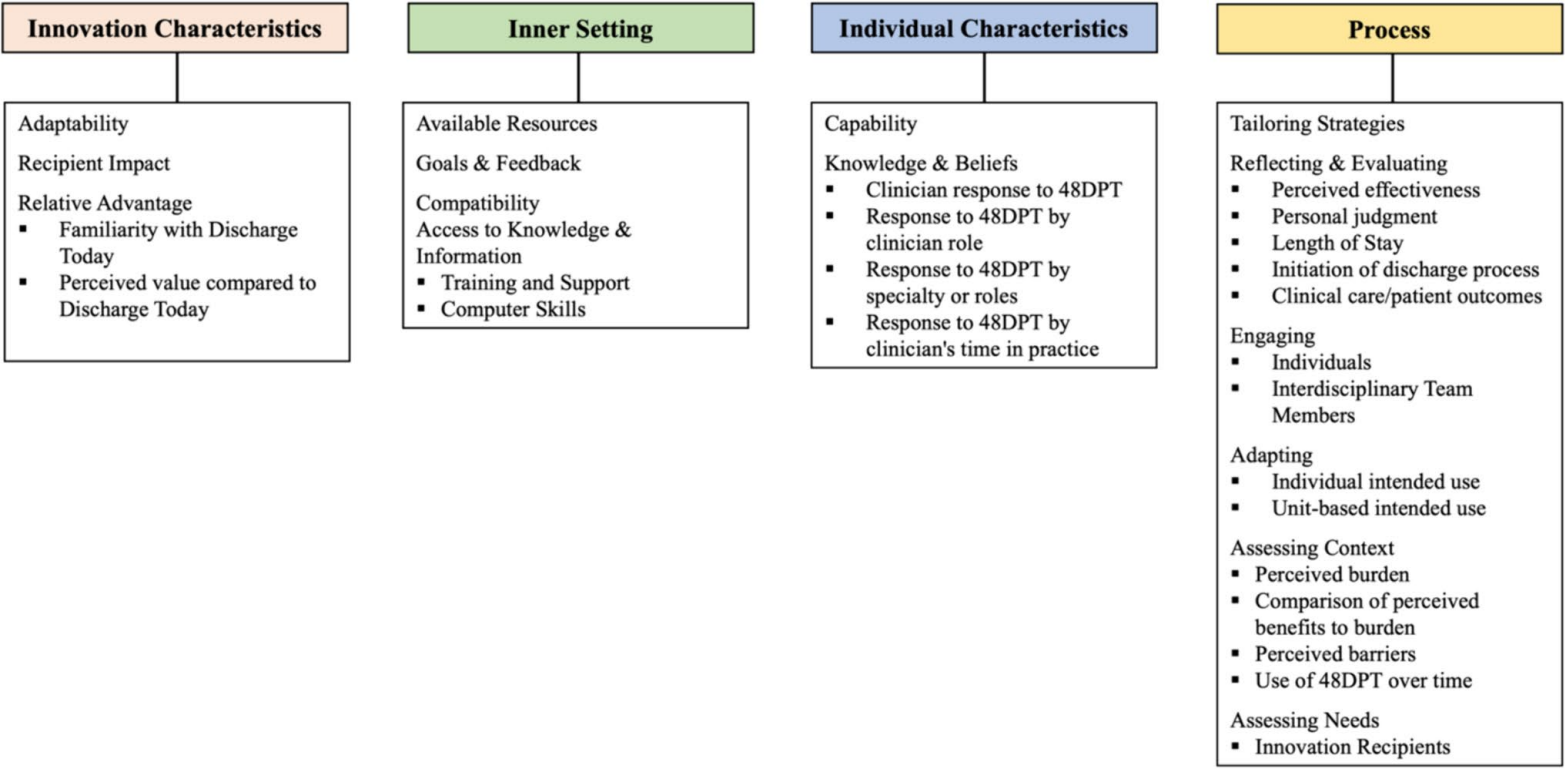
Concept map of CFIR constructs and subconstructs by domain

**Fig. 3:**
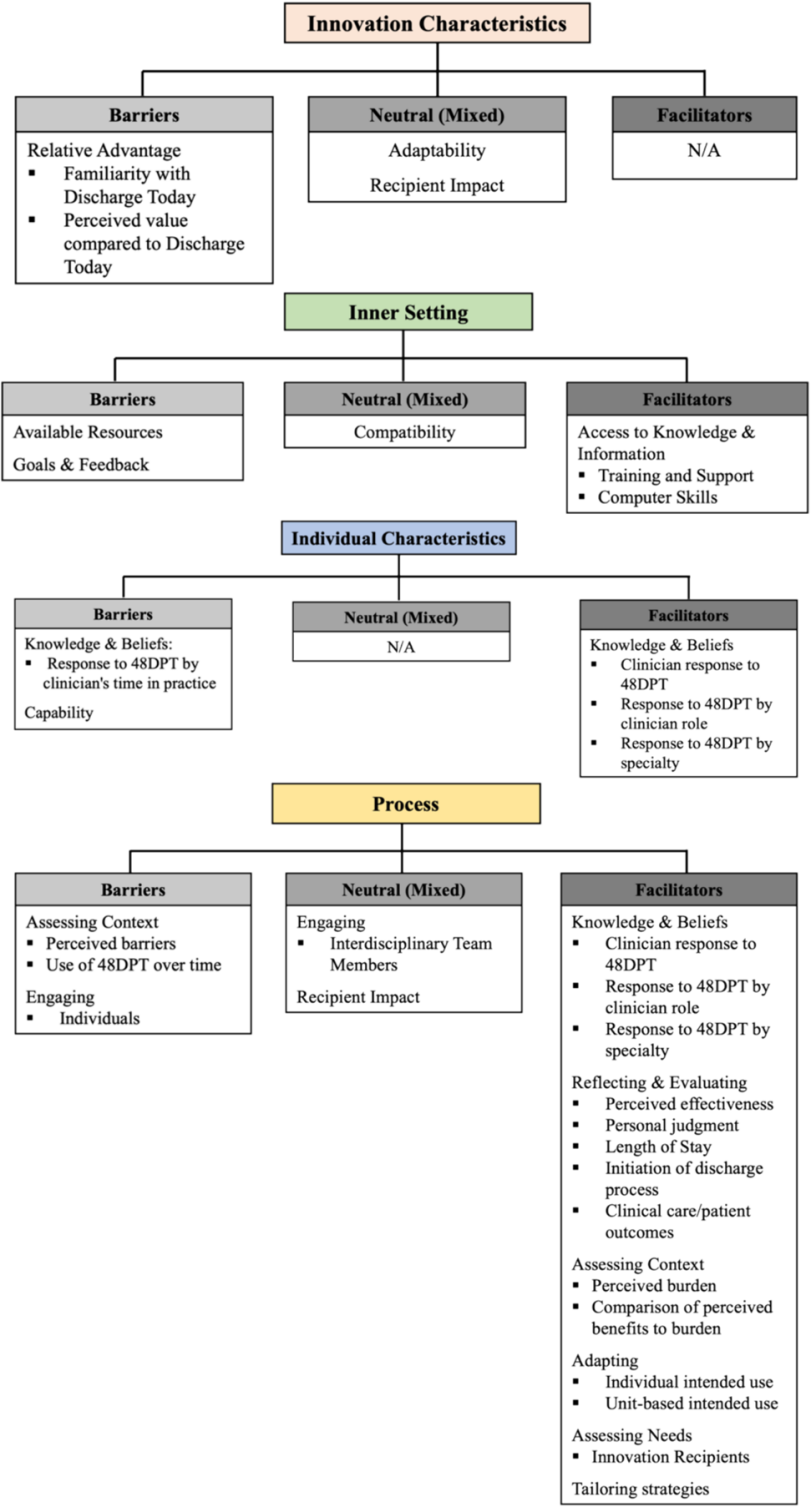
CFIR domains, constructs and subconstructs related to barriers, neutral (mixed), and facilitators

This is the first study that systematically evaluated the implementation process of internally developed AI-assisted EHR CDS aimed at predicting discharge date using a combination of implementation science frameworks RE-AIM and CFIR. Previous studies utilized qualitative interviews for assessing challenges to AI implementation in healthcare without aligning their analyses with existing implementation science frameworks [55–57]. The addition of widely recognized dimensions and constructs from implementation science facilitates comparison between various studies and different implementation programs [58, 59]. The results of our study are well aligned with the previous findings emphasizing the crucial importance of sociotechnical factors and specific workflow contexts for long-term success of EHR CDS implementation and its impact on routine clinical care [60, 61]. The systematic assessment of the 48DPT implementation allowed the identification of facilitators and barriers to implementation, such as lack of awareness, lack of accuracy, limited accessibility, and transparency. These findings are summarized in Figs. 1, 2, 3. Based on our evaluation, the factors that are crucial for the successful implementation of AI-assisted EHR CDS are presented in Table 8. Future implementation efforts of AI-assisted EHR CDS should engage the key clinical stakeholders in the AI tool development from the very inception of the project, support transparency and explainability of the AI models, provide ongoing education and onboarding of the clinical users, and obtain continuous input from clinical staff on the CDS performance.

**Table 8:** Criteria for successful implementation of AI-assisted EHR CDS

Criteria	Action
Early engagement	A broad spectrum of clinical key stakeholders are involved from the project commencement
Ongoing education	Training of major AI concepts, biases, and limitations is provided to enhance clinical practice
Stepwise implementation	The project begins with small-scale implementation to allow for gradual adaptations to the AI-assisted CDS
Clinician autonomy	Providers are explained that the AI-assisted CDS is aimed at assisting not replacing their judgement
Explainability and transparency	Trust is facilitated by providing clear explanations and visualizations of internal workings of the AI-assisted EHR CDS
User-focused design	The CDS interface should be informed by clinicians’ input
Continuous user input	Ongoing collection of clinicians’ feedback and suggestions
Success use cases	Successful examples of the AI-assisted CDs use are promulgated via rounds and institutional media
Support from leadership	Institutional leadership is essential for long-term sustainability
Cultural change	Culture of innovation and continuous learning is supported and encouraged

## Data Availability

No datasets were generated or analysed during the current study.

## Appendix 1

Moderator Guide for Semi-Structured Qualitative Interviews

Respondent Characteristics:

1. Primary unit(s) _____________________
2. Role (Case manager, Social Worker, Nurse Manager, Unit Medical Director, Attending, Intern/Resident, Nurse Practitioner, Physician Assistant) _____________________________________________________________________
3. How long have you worked at MSH? (0–1 year, 2–4 years, 5–10 years, > 10 years) _____________________________________________________________________

**Script:** State that 48DPT refers to the used during morning interdisciplinary rounds on Mount Sinai wards to predict whether patients will be ready for discharge within 48 h.

**Table:**

*RE-AIM Dimension*	*Questions*
Reach (individual level)	**For Unit-based Leadership (Case Manager, Social Worker, Nurse Manager, Unit Medical Director):** On a typical weekday, How often does at least 1 member of the interdisciplinary team review the’s result (i.e., ready or not ready for discharge in 48 h)? Are there clinician groups for whom the is less likely to be used, such as from certain departments or teams? If yes, please describe Does use of the 48DPT vary by any patient factors, such as age, race, or disease? If yes, please describe **For Clinicians (Attending, Intern/Resident, Nurse Practitioner, Physician Assistant):** Are you aware that an electronic is in use, called the 48DPT, to help predict which patients may be able to be discharged within 48 h? On a typical weekday, how often does at least 1 member of the interdisciplinary team review the 48DPT’s result (i.e., ready or not ready for discharge in 48 h)? Does use of the 48DPT vary by any patient factors, such as age, race, or disease? If yes, please describe
Efficacy / effectiveness (individual level)	How effective is 48DPT in identifying medical readiness for discharge? How often is discharge better predicted by 48DPT as compared to your judgment? Do you feel the 48DPT helps decrease LOS? If yes, please describe Do you feel the 48DPT helps initiate the processes needed for discharge (such as scheduling appointments), earlier? If yes, please describe Do you feel the 48DPT improves clinical care or patient outcomes? If yes, please describe Are you familiar with the “Discharge Today” field in Epic? If yes, do you feel the 48DPT and Discharge Today tool are redundant to each other, or do you feel each offers unique information? If you feel they are redundant, which of these 2 tools do you feel is more valuable? Why?
Adoption (setting and/or organizational level)	**For Case Managers, Social Workers, Nurse Managers:** Is 48DPT used as intended? If not, tell us how the use varies How do clinicians react when you or another team member mentions that a discharge is predicted in 48 h? Does the reaction vary by the clinician’s role (NPs, PAs, Attendings, Hou staff)? If yes, please describe Does the response to the 48DPT, such as you or another team member stating that discharge is predicted in 48 h, vary according to specialty (Medicine, Fam Med, etc.), roles (attending, resident, NP, PA)? If yes, please describe Does the response to the 48DPT, such as you or another team member stating that discharge is predicted in 48 h, vary according to clinician’s time in practice? If yes, please describe **For Clinicians (Attending, Intern/Resident, Nurse Practitioner, Physician Assistant):** Is 48DPT used as intended? If not, tell us how the use varies Does the use of the 48DPT vary by unit? If yes, tell us how use varies
Implementation (setting and/or organizational level)	Were you involved in developing the process for the use of the 48DPT? Do you feel that the members of the interdisciplinary team, such as CM, SW, Nurse Managers, and the clinicians, were involved adequately in developing the process for the use of the 48DPT? Do you feel 48DPT was integrated well into workflow? If not, how could integration have been improved? What training and support services are needed by 48DPT users? What level of computer skills are needed to use the 48DPT?
Maintenance (individual and setting levels)	How much of a burden is using the 48DPT for you? Do you feel the benefits of the 48DPT outweigh the burden? What are the main barriers to use of the 48DPT? Have there been any efforts by leaders to receive feedback on the use of the 48DPT in order to make revisions? If yes, please describe Has the use of 48DPT changed over time? If yes, please describe Have any s helped you improve your use of the 48DPT (such as training sessions, individual feedback, unit-based metrics) If yes, please describe How could use of the 48DPT be improved?

## Appendix 2

Table 9

**Table 9:** RE-AIM guided interview questions with CFIR domains and constructs

Interview questions by RE-AIM dimension	Stakeholder group(s)	CFIR domains	CFIR constructs	Subconstruct
Reach				
Are you aware that an electronic is in use, called the 48DPT, to help predict which patients may be able to be discharged within 48 h?	Clinician	Individuals	Capability	
Are there clinician groups for whom the is less likely to be used, such as from certain departments or teams? If yes, please describe	Unit-based leadership	Process	Tailoring Strategies	
On a typical weekday, how often does at least 1 member of the interdisciplinary team review the 48DPT’s result (i.e., ready or not ready for discharge in 48 h)?	Unit-based leadership, Clinicians	Innovation outcomes	Innovation Recipient Impact	
Does use of the 48DPT vary by any patient factors, such as age, race, or disease? If yes, please describe	Unit-based leadership, Clinicians	Innovation	Innovation Adaptability	
**Efficacy/effectiveness**				
How effective is 48DPT in identifying medical readiness for discharge?	Unit-based leadership, Clinicians	Process	Reflecting & Evaluating	Perceived effectiveness
How often is discharge better predicted by 48DPT as compared to your judgment?	Unit-based leadership, Clinicians	Process	Reflecting & Evaluating	Personal judgment
Do you feel the 48DPT helps decrease LOS? If yes, please describe	Unit-based leadership, Clinicians	Process	Reflecting & Evaluating	Length of Stay
Do you feel the 48DPT helps initiate the processes needed for discharge (such as scheduling appointments), earlier? If yes, please describe	Unit-based leadership, Clinicians	Process	Reflecting & Evaluating	Initiation of discharge process
Do you feel the 48DPT improves clinical care or patient outcomes? If yes, please describe	Unit-based leadership, Clinicians	Process	Reflecting & Evaluating	Clinical care/Patient outcomes
Are you familiar with the “Discharge Today” field in Epic? If yes, do you feel the 48DPT and Discharge Today s is redundant to each other, or do you feel each offers unique information?	Unit-based leadership, Clinicians	Innovation	Relative Advantage	Familiarity with Discharge Today
If you feel they are redundant, which of these 2 s do you feel is more valuable? Why?	Unit-based leadership, Clinicians	Innovation	Relative Advantage	Perceived value compared to Discharge Today
**Adoption**				
Is 48DPT used as intended? If not, tell us how the use varies	Unit-based leadership, Clinicians	Process	Adapting	Individual intended use
Does the use of the 48DPT vary by unit? If yes, tell us how use varies	Clinicians	Process	Adapting	Unit-based intended use
How do clinicians react when you or another team member mentions that a discharge is predicted in 48 h?	Unit-based leadership	Individuals	Knowledge & Beliefs	Clinician response to 48DPT
Does the reaction vary by the clinician’s role? If yes, please describe	Unit-based leadership	Individuals	Knowledge & Beliefs	Response to 48DPT by clinician role
Does the response to the 48DPT, such as you or another team member stating that discharge is predicted in 48 h, vary according to specialty, roles? If yes, please describe	Unit-based leadership	Individuals	Knowledge & Beliefs	Response to 48DPT by specialty or roles
Does the response to the 48DPT, such as you or another team member stating that discharge is predicted in 48 h, vary according to clinician’s time in practice? If yes, please describe	Unit-based leadership	Individuals	Knowledge & Beliefs	Response to 48DPT by clinician's time in practice
**Implementation**				
Were you involved in developing the process for the use of the 48DPT?	Unit-based leadership, Clinicians	Process	Engaging	Individuals
Do you feel that the members of the interdisciplinary team (e.g., Case Managers, Social Workers, and the clinicians) were involved adequately in developing the process for the use of the 48DPT?	Unit-based leadership, Clinicians	Process	Engaging	Interdisciplinary Team Members
Do you feel 48DPT was integrated well into workflow? If not, how could integration have been improved?	Unit-based leadership, Clinicians	Inner Setting	Compatibility	
What training and support services are needed by 48DPT users? (Enabling)	Unit-based leadership, Clinicians	Inner Setting	Access to Knowledge & Information	Training and Support
What level of computer skills are needed to use the 48DPT?	Unit-based leadership, Clinicians	Inner Setting	Access to Knowledge & Information	Computer Skills
**Maintenance**				
How much of a burden is using the 48DPT for you?	Unit-based leadership, Clinicians	Process	Assessing Context	Perceived burden
Do you feel the benefits of the 48DPT outweigh the burden?	Unit-based leadership, Clinicians	Process	Assessing Context	Comparison of perceived benefits to burden
What are the main barriers to use of the 48DPT?	Unit-based leadership, Clinicians	Process	Assessing Context	Perceived barriers
Have there been any efforts by leaders to receive feedback on the use of the 48DPT in order to make revisions? If yes, please describe	Unit-based leadership, Clinicians	Inner Setting	Goals & Feedback	
Has the use of 48DPT changed over time? If yes, please describe	Unit-based leadership, Clinicians	Process	Assessing Context	
Have any s helped you improve your use of the 48DPT (such as training sessions, individual feedback, unit-based metrics) If yes, please describe	Unit-based leadership, Clinicians	Inner Setting	Available Resources	
How could use of the 48DPT be improved?	Unit-based leadership, Clinicians	Assessing Needs	Innovation Recipients	

## Appendix 3

Table 10

**Table 10:** Ratings assigned to CFIR construct by Stakeholder Role

		Clinicians		Unit-based Leadership				
CFIR Construct and Sub-constructs	All Roles	Hospitalist/ Attending	Attending/ Unit Medical Director	Unit Medical Director	Case Manager	Nurse Manager		Social Worker
**INTERVENTION**								
Relative Advantage: Familiarity with Discharge Today	**-1**	-2	-2	-1	-1	1	-1	
Relative Advantage: Perceived value compared to Discharge Today	**-1**	-2	-1	2	-2	0	-1	
Innovation Adaptability	**0**	0	M	0	0	0	0	
**INNER SETTING**								
Compatibility	**0**	0	0	2	-2	-1	0	
Available Resources	**-1**	M	-1	-1	-1	-1	-1	
Access to Knowledge & Information: Training and Support	**1**	-1	0	0	1	M	1	
Access to Knowledge & Information: Computer Skills	**2**	M	2	2	2	M	2	
Goals & Feedback	**-2**	M	-2	-2	-2	-2	-2	
**INDIVIDUAL**								
Knowledge & Beliefs: Clinician response to 48DPT	**1**	M	M	1	0	2	1	
Knowledge & Beliefs: Response to 48DPT by clinician role	**1**	M	M	1	-1	0	1	
Knowledge & Beliefs: Response to 48DPT by specialty or roles	**1**	M	M	M	1	0	2	
Knowledge & Beliefs: Response to 48DPT by clinician's time in practice	**-1**	M	M	-1	-1	-1	0	
Capability	**-2**	-2	-1	M	M	M	M	
**PROCESS**								
Assessing Needs: Innovation Recipients	**1**	1	1	1	1	-1	1	
Assessing Context: Perceived burden	**2**	M	2	2	-1	2	2	
Assessing Context: Comparison of perceived benefits to burden	**2**	M	2	2	-1	2	2	
Assessing Context: Perceived barriers	**-2**	M	-2	-1	-2	-2	-2	
Assessing Context: Use of 48DPT over time	**-1**	M	-1	-1	-2	0	-1	
Engaging: Individuals	**-1**	-1	-1	-1	-1	1	-1	
Engaging: Interdisciplinary Team Members	-2	-1	-2	-2	-2	-2	-2	
Reflecting & Evaluating: Perceived effectiveness	**1**	0	2	1	1	0	1	
Reflecting & Evaluating: Personal judgment	**1**	0	1	1	-1	0	1	
Reflecting & Evaluating: Length of Stay	**1**	0	2	1	-1	1	1	
Reflecting & Evaluating: Initiation of discharge process	**1**	N/A	2	1	-1	1	1	
Reflecting & Evaluating: Clinical care/ Patient outcomes	**1**	N/A	2	1	-1	0	1	
Adapting: Individual intended use	**1**	0	2	1	1	1	1	
Adapting: Unit-based intended use	**1**	0	2	N/A	N/A	N/A	N/A	
Tailoring Strategies	**1**	N/A	N/A	1	0	1	0	
**OUTCOMES**								
Innovation Recipient Impact	**0**	-2	1	1	-1	-1	1	
